# A Randomized Pilot Trial to Evaluate the Bioavailability of Natural versus Synthetic Vitamin B Complexes in Healthy Humans and Their Effects on Homocysteine, Oxidative Stress, and Antioxidant Levels

**DOI:** 10.1155/2019/6082613

**Published:** 2019-12-12

**Authors:** Meinrad Lindschinger, Franz Tatzber, Wolfgang Schimetta, Irene Schmid, Barbara Lindschinger, Gerhard Cvirn, Olaf Stanger, Eugenia Lamont, Willibald Wonisch

**Affiliations:** ^1^Institute of Nutritional and Metabolic Diseases, Outpatient Clinic Laßnitzhöhe, Laßnitzhöhe, Austria; ^2^Otto Loewi Research Center for Vascular Biology, Immunology and Inflammation, Chair of Immunology and Pathophysiology, Medical University of Graz, Graz, Austria; ^3^Department of Applied Systems Research and Statistics, Johannes Kepler University, Linz, Austria; ^4^Otto Loewi Research Center for Vascular Biology, Immunology and Inflammation, Chair of Physiological Medicine, Medical University of Graz, Graz, Austria; ^5^Helios Clinic Munich West, Clinic for Vascular and Endovascular Surgery, Munich-Pasing, Germany; ^6^Clinic Section for Surgical Research Department of Surgery Medical University of Graz, Graz, Austria

## Abstract

The vitamin B complex comprises 8 different water-soluble constituents that humans must sequester from the diet. This pilot study compared natural versus synthetic vitamin B complexes for their bioavailability, accumulation, and their impact on antioxidants, homocysteine levels, and oxidative stress. We conducted a double-blind randomized clinical trial with thirty healthy participants. They were randomly assigned to group N (natural) and group S (synthetic). Vitamin B was ingested daily for 6 weeks in the range of about 2.5 times above the recommended daily allowance. Blood samples were taken at baseline, 1.5 h, 4 h, 7 h (diurnal), 6 w (discontinuation of supplements), and 8 w (washout). Blood levels of thiamine (B_1_), riboflavin (B_2_), pyridoxine (B_6_), folic acid (B_9_), cobalamin (B_12_), homocysteine, total antioxidants, peroxidase activity, polyphenols, and total peroxides were determined. Compared to initial values, serum levels of each B vitamin increased at the end of the supplementation period: i.e., B_1_ (+23% N; +27% S), B_2_ (+14% N; +13% S), B_6_ (+101% N; +101% S), B_9_ (+86% N; +153% S), and B_12_ (+16% N) (*p* < 0.05). Homocysteine (-13% N) decreased, while peroxidase activity (+41% S) and antioxidant capacity increased (+26% N). Short-term effects were already observed after 1.5 h for B_9_ (+238% N; +246% S) and after 4 h for vitamin B_2_ (+7% N; +8% S), B_6_ (+59% N; +51% S), and peroxidase activity (+58% N; +58% S). During the washout period, serum levels of B vitamins decreased except for thiamine and peroxidase activity, which increased further. This clinical pilot study revealed comparable bioavailability for both natural and synthetic B vitamins but did not show statistically noticeable differences between groups despite some favourable tendencies within the natural vitamin group, i.e., sustained effects for cobalamin and endogenous peroxidase activity and a decrease in homocysteine and oxidative stress levels.

## 1. Introduction

The vitamin B complex comprises eight water-soluble constituents: thiamine (B_1_), riboflavin (B_2_), niacin (B_3_), pantothenic acid (B_5_), pyridoxine (B_6_), biotin (B_7_), folic acid (B_9_), and cobalamin (B_12_). They are functionally related and cooperate in protein, lipid, and nucleic acid synthesis, energy production, and immune defence; they also exert numerous effects on brain function. These essential nutrients are sequestered from the diet and act as coenzymes in various processes. Generally, B vitamins are synthesized in plants with one exception, cobalamin, which is abundant in red meat and is synthesized by bacteria [1, 2].

The composition of the vitamin B complex is distinctive, and combined supplementation requires a particular proportion for each component. Unbalanced intake might deplete other vitamins and disturb metabolism [3]. Vitamin B from plants contains diverse variants of a single vitamin, e.g., vitamin B_6_ and its vitamers pyridoxamine 5′-phosphate and pyridoxal 5′-phosphate [4] with synergistic effects, and thus differs from a synthetic vitamin B. Little is known about bioavailability and potential health benefits of natural versus synthetic B vitamins. Natural lipid-soluble tocopherol (vitamin E) has been shown to have a twofold higher availability compared to synthetic vitamin E [5]; this was also the case for natural phenolic antioxidants vs. synthetic compounds [6, 7]. In contrast, the bioavailability of synthetic vitamin C was comparable to vitamin C derived from kiwifruit [8].

Each vitamin B was shown to participate in the antioxidative response against oxidative stress [9–12], most notable expressed as degradation of homocysteine [13]. Oxidative stress is defined as an imbalance between pro- and antioxidative mechanisms in favour of an excess of reactive oxygen species, which damage lipids, proteins, carbohydrates, and nucleic acids. Yet, the negative effect can be prevented by antioxidants. The combined vitamin B supplementation causes a marked decrease in ROS, protein oxidation, and an enhancement of antioxidative enzymes [9, 14–16]. Subjects continuously exposed to low-grade oxidative stress, such as manual laborers [17] and patients suffering from schizophrenia [18] or chronic illnesses including cancer, might particularly benefit from the antioxidant and anti-inflammatory impact of the vitamin B in the prevention of lipid peroxidation [19, 20].

These considerations led us to design a double-blind randomized crossover trial to evaluate the bioavailability and health benefits of natural versus synthetic B vitamins in healthy subjects. We hypothesized that in counteracting oxidative stress, natural vitamin B complex is at least as good or better than the synthetic vitamin with respect to bioavailability, accumulation, and storage effects, as well as the impact on antioxidative mechanisms.

## 2. Material and Methods

### 2.1. Study Participants

Thirty healthy participants were enrolled at the Institute of Nutritional and Metabolic Diseases in the Outpatient Clinic Laßnitzhöhe. The blood (max. 20 mL) was drawn from an antecubital vein from the seated subject. Exclusion criteria were poor compliance (<80% of natural and/or synthetic vitamin B supplement intake during the length of study), pregnancy or lactation, chronic infections, cholesterol > 240 mg/dL, consumption of vitamins, trace elements, or fatty acid supplements or participation in any other clinical trials within the past three months, vegan diet, consumption of more than 0.5 L beer per day, impaired kidney and/or liver function, and known chronic diseases including cardiovascular disease, cancer, psychosis, diabetes mellitus, and autoimmune diseases. One subject withdrew from the study after an adverse reaction (flush) upon first taking the investigated product.

### 2.2. Study Design

The experimental design was a randomized, crossover double-blind pilot study. The study was conducted in accordance with the Declaration of Helsinki and was approved by the local ethics committee on 22^nd^ of March 2017 (EK 29-271 ex 16/17) (Clinical Trial Registration URL: http://www.clinicaltrials.gov. Unique identifier: NCT03444155). We regrettably only applied for a clinical trial number after we had begun to recruit patients. It was our belief that all we needed at that point was the votum from the Ethics Commission, which we had. We rectified this error as soon as it came to our attention and will avoid such an omission in the future. The authors confirm that all ongoing and related trials for this drug/intervention are registered.

**Figure 1:**
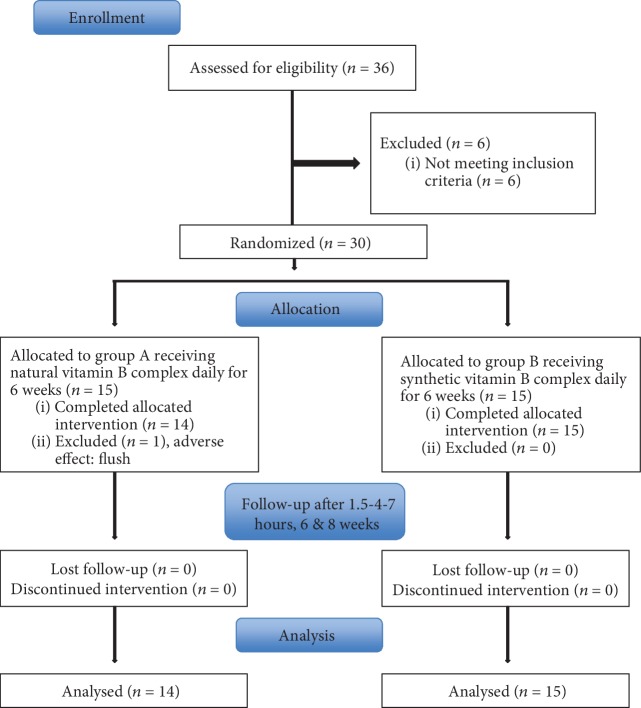
CONSORT flow diagram of healthy subjects supplemented daily with a natural or synthetic vitamin B complex for 6 weeks and a washout period for 2 weeks, according to the CONSORT 2010 Statement.

All participants provided written informed consent. The study was performed between May 8, 2017 (FPI) and October 3, 2017 (LPO).  Figure 1 shows the CONSORT flow diagram, according to the CONSORT 2010 Statement [21].

### 2.3. Procedure

Following a three-week screening period, thirty participants were randomized and enrolled (1 : 1 ratio—group N vs. group S; using a validated system) to receive either a natural vitamin B complex (Panmol B complex) or a synthetic vitamin B complex with identical concentrations over a 6-week treatment period and a washout period of 2 weeks. The contents of each single vitamin B of the synthetic B complex (LOT-Nr. L17050078 MHD 05.2019) were equivalent to those of the Panmol B complex. The Panmol B complex (LOT-Nr. L17050077) was manufactured by Vis Vitalis GmbH (Salzburg, Austria) and is commercially available as PANMOL® B complex. The synthetic vitamin B complex was manufactured in the laboratory of Vis Vitalis GmbH (Salzburg, Austria), and since it is not commercially available, the raw materials and manufacturing processes for the synthetic vitamin B complex are described in detail.

### 2.4. Raw Materials for the Synthetic Vitamin B Complex

The following are the raw materials for the synthetic vitamin B complex: (1) thiamine hydrochloride, DSM Nutritional Products Europe, Basel, Switzerland; (2) riboflavin, DSM Nutritional Products Europe, Basel, Switzerland; (3) nicotinamide, DSM Nutritional Products Europe, Switzerland; (4) calcium-D-pantothenate, Productos Químicos Gonmisol SA, Barcelona, Spain; (5) pyridoxine hydrochloride, DSM Nutritional Products Europe, Basel, Switzerland; (6) biotin premix 1%, Rieser GmbH, Mattersburg, Austria; (7) folic acid premix 1%, Rieser GmbH, Mattersburg, Austria; and (8) cyanocobalamin, DSM Nutritional Products Europe, Basel, Switzerland.

The following are the manufacturing processes for the synthetic vitamin B complex: HV-01-001V1 General Manufacturing Instructions:

1. All raw materials are prepared and tested
2. Before mixing, the temperature and the relative humidity are recorded and documented in the batch record
3. All devices used are cleaned with the appropriate disinfectants
4. Amounts of ingredients up to 1000 g per batch are mixed in the Department of Galenics (Prodima AC-MJ 50 or Rhönrad mixer). The mixer used is noted in the batch record
5. Ingredients of more than 1000 g per batch are weighed in the Mixing Section
6. Mixing is performed according to SOP-05-005 (Sama, Drais, Prodima AC-LI500S mixer)
7. The net weight of the batch is documented in the batch record (powder mixtures). For products stored in white tubs for further processing (mixing in Drais and Prodima), tare/gross weights are documented in the mixing log
8. Deviations from manufacturer's instructions for sensitive formulations/special formulations, and recipes for which the general manufacturing instructions for mixing do not apply, are documented in our own manufacturing specifications

Basic characteristics are listed in Table 1.

**Figure 2:**
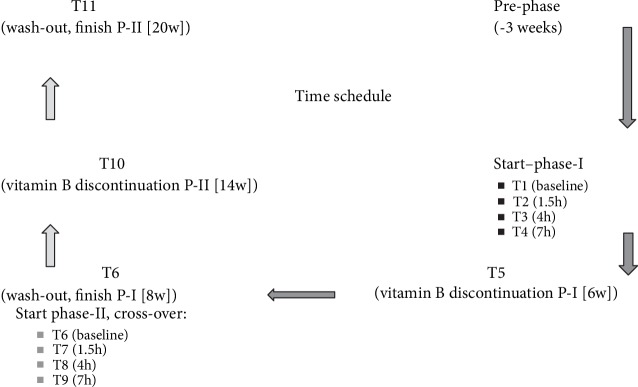
Study design. Time schedule for blood sampling (T1-T11) and supplementation with vitamin B complex (natural vs. synthetic) applicable to both study groups. The study was initiated with a three-week run-in phase before the first study day. After study onset, blood samples for information on the diurnal cycle were taken at baseline (T1), 1.5 hours after the first ingestion of the vitamin B complex (T2), 4 hours after the first ingestion (T3), and 7 hours after the first ingestion (T4). Supplementation was terminated after a period of 6 weeks (T5) followed by a 2-week washout period (T6), to complete the first phase of this crossover study. This timepoint was simultaneously the baseline for the second study phase. The vitamin B complex was crossed over in the two groups (natural vs. synhtetic and *vice versa*), and blood samples were taken after 1.5 hours after ingestion of the first substituted vitamin B complex (T7), then after 4 hours (T8) and 7 hours (T9). Supplementation in the second study phase was terminated after a period of 6 weeks (T10), followed by a washout period of another 6 weeks (T11) at the end point of the study. Due to carryover effects in the majority of biomarkers, crossover comparisons were generally dispensed with, resulting in a restriction to group comparisons in phase 1, as emphasized by dark arrows.

Clinical assessments were performed at baseline (T1), 1.5 hours after the first ingestion of vitamin B supplements (T2), 4 hours (T3), 7 hours (T4), week 6—discontinuation of the supplements (T5), and week 8—washout period I (T6). The last examination in the first period, i.e., T6, was the baseline determination for the second period as well, in a crossover design. In the second period, blood samples were again taken at 1.5 hours after the first ingestion of the crossed over B complex supplementation (T7), 4 hours (T8), 7 hours (T9), week 14—discontinuation of supplements (T10), and after another 6 weeks, i.e., week 20—at the end of washout period II (T11)—for details, see Figure 2.

Study medication was manufactured as hydroxypropyl methylcellulose capsules (size 0, white coloured). Capsules and packaging were identical for natural and synthetic vitamin B complexes. Each package contained 126 capsules per subject and period. Patients took 3 capsules by mouth in the morning with 250 mL of water. Each portion (3 capsules) contained vitamin B_1_ (thiamine—2.93 mg), vitamin B_2_ (riboflavin—3.98 mg), vitamin B_3_ (niacin—29.85 mg), vitamin B_5_ (pantothenic acid—10.95 mg), vitamin B_6_ (pyridoxine—3.38 mg), vitamin B_7_ (biotin—0.108 mg), vitamin B_9_ (folic acid—0.69 mg), and vitamin B_12_ (cobalamin—8.85 *μ*g). Study medication was dispensed at T1 and T6, and was self-administered by the participants, who had received dosage instructions. The investigators and personnel involved in monitoring and data handling were blinded to the study medication to maintain the double-blind condition.

### 2.5. Analysis of B Vitamins in Human Samples

#### 2.5.1. Thiamine (Vitamin B_1_)

Blood thiamine levels were determined with a commercially available high-performance liquid chromatography (HPLC) reagent kit from Chromsystems (Munich, Germany). Briefly, after preliminary derivatisation of the blood samples, aliquots of 50 *μ*L were injected into the HPLC and analysed with a flow rate of 1.0 mL/min at room temperature. Fluorescence was detected at an excitation wavelength of 367 nm (Ex 367 nm) versus an emission wavelength of 435 nm (Em 435 nm).

#### 2.5.2. Riboflavin (Vitamin B_2_)

Blood riboflavin concentrations were determined with a commercially available HPLC reagent kit from Recipe Chemicals and Instruments Ltd. (Munich, Germany). After derivatisation of the whole blood, 50 *μ*L of the samples was injected into the reversed-phase HPLC column with fluorometric detection at a wavelength of Ex 450 nm vs. Em 530 nm.

#### 2.5.3. Pyridoxine (Vitamin B_6_)

Blood pyridoxine concentrations were determined with a commercially available HPLC reagent kit provided by Recipe Chemicals and Instruments Ltd. (Munich, Germany). After derivatisation of the whole blood samples, 50 *μ*L of aliquots was injected into the reversed-phase HPLC column with fluorometric detection at a wavelength of Ex 370 nm vs. Em 470 nm.

#### 2.5.4. Folic Acid (Vitamin B_9_)

Plasma folate was measured with the ARCHITECT® folate assay (Abbott Laboratories, Abbott Park, USA), a chemiluminescent microparticle folate-binding protein assay.

#### 2.5.5. Cobalamin (Vitamin B_12_)

Cobalamin was quantified with a chemiluminescent microparticle intrinsic factor assay with the ARCHITECT® B12 assay from Abbott Laboratories (Abbott GmbH & CoKG, Max-Planck-Ring 2, 65205 Wiesbaden, Germany).

#### 2.5.6. Homocysteine (tHcy)

Total L-homocysteine was determined with the Architect Homocysteine® assay (Abbott GmbH, see above) according to the manufacturer's instructions. This method uses CMIA technology in a one-step immunoassay to detect total L-homocysteine in plasma and serum.

#### 2.5.7. Total Antioxidant Capacity (TAC®)

Total antioxidant capacity was determined with a commercially available colorimetric method supplied by LDN (Labor Diagnostic Nord, Nordhorn, Germany). This method is based on inhibition of a peroxide/peroxidase reaction using 3,5,3′5′-tetramethylbenzidine (TMB) as substrate. Antioxidants in plasma or serum samples inhibit the attack of reactive oxygen species (ROS) on TMB that is associated with a quenching of the colorimetric signal. After 20 minutes of incubation at 4°C, the blue colour changes to yellow upon addition of the stop solution. Absorbance was read at 450 nm (reference wavelength 620 nm).

#### 2.5.8. Peroxidase Activity (EPA®)

Endogenous peroxidase activity was determined according to the method of Tatzber et al. [22] with a commercially available colorimetric method purchased from LDN (Labor Diagnostic Nord, Nordhorn, Germany), a dual method for the measurement of both peroxides and peroxidase activity.

#### 2.5.9. Total Peroxides (TOC®)

Peroxides were likewise determined with a colorimetric assay supplied by LDN (Labor Diagnostic Nord, Nordhorn, Germany). Peroxides in serum and plasma samples react with horseradish peroxidase to produce a blue-green substrate cation (TMB). After addition of the stop solution, the colour changes to yellow and is measured at a wavelength of 450 nm (ref. 620 nm). A linear standard curve (up to 1 mM) was used for quantification.

#### 2.5.10. Polyphenols Microtitre (PPm®)

Total polyphenol content in serum samples was determined with a commercially available kit from Omnignostica Ltd. (Höflein/D., Austria). This method was described previously [17] and is based on the reaction of polyphenols with transition metals that occurs when the Folin-Ciocalteu reagent is used. The dark-coloured complex was measured at 766 nm. Serial dilutions of gallic acid were used as a standard curve according to the manufacturer's instructions.

### 2.6. Outcomes

The primary outcome was the bioavailability and accumulation of serum vitamin B_1_, B_2_, B_6_, B_9_, and B_12_ in group N (natural vitamin B complex) and group S (synthetic vitamin B complex). Secondary outcome was the impact of natural and synthetic vitamin B complex on antioxidative mechanisms (TAC, EPA, PPm) to counteract oxidative stress (tHcy, TOC).

### 2.7. Sample Size Determination

The chosen sample size of 30 participants with a postulated drop-out rate of 20% and a crossover approach should provide general initial insights into a possibly better bioavailability and more sustainable efficacy of natural B vitamins compared to synthetic B vitamins in this pilot study.

### 2.8. Allocation and Blinding

The study participants were randomly assigned 1 : 1 to the two groups (natural vitamins in phase 1 and synthetic vitamins in phase 2 vs. synthetic vitamins in phase 1 and natural vitamins in phase 2). The software R package‚ blockrand (Greg Snow. blockrand: Randomization for block random clinical trials. R package version 1.3. Published 2013-01-18. https://CRAN.R-project.org/package=blockrand), was used by the statistician to create random sequences in block sizes of 2 and 4.

The subjects were given consecutive trial subject numbers in the order of their inclusion in the study. For every trial subject number, two packages with investigational products (one package for phase 1 and one package for phase 2) were prepared and provided. Until the database was closed, only the statistician and the team responsible for the preparation of the blinded investigational products (Vis Vitalis GmbH, Salzburg, Austria) knew the group assignment, but were bound to secrecy against the third parties. The study participants, the trial staff, and the data manager were blinded throughout the study and thereafter until the completion of all outstanding issues. Emergency information was present in sealed opaque envelopes containing single decodings, but none of these envelopes was opened. All capsules used in the study were identical as to shape, size, taste, color, and smell, so the investigational products were indistinguishable.

### 2.9. Statistical Analysis

A crossover study was originally planned, but 3 of the 5 vitamins and 3 of the 5 biomarkers had to be excluded from a crossover evaluation due to carryover effects. Of the remaining crossover analyses, almost half were unusable due to period effects. We accordingly dispensed with crossover comparisons general, which in turn limited group comparisons to phase 1.

All data sets of continuous variables were checked for normal distribution (Kolmogorov-Smirnov test with Lilliefors significance correction, type I error = 10%). Groups of normally distributed data sets were compared with the *t*-test for independent samples (test for variance homogeneity: Levene test, type I error = 5%). Data sets of continuous variables without normal distribution were compared with the exact Mann-Whitney *U* test. Fisher's exact test was used to compare of sex, the only nominal variable.

The conciseness of the course of vitamin and biomarker blood levels within the groups was investigated by two-sided 95% confidence intervals of differences between two examinations.

Since the type I error was not adjusted for multiple testing, the results of inferential statistics are descriptive only and the use of the term “significant” in the description of the study results always reflects only a local *p* < 0.05 but no error probability below 5%. Statistical analysis was performed with the open-source R statistical software package, version 3.4.1 (The R Foundation for Statistical Computing, Vienna, Austria).

## 3. Results

### 3.1. Study Participants

Thirty healthy individuals participated in the study between May 8, 2017 and October 3, 2017. One subject was excluded from the study after an adverse reaction (flush) upon first ingestion of the investigated product (Figure 1).

### 3.2. Thiamine (Vitamin B_1_)

**Figure 3:**
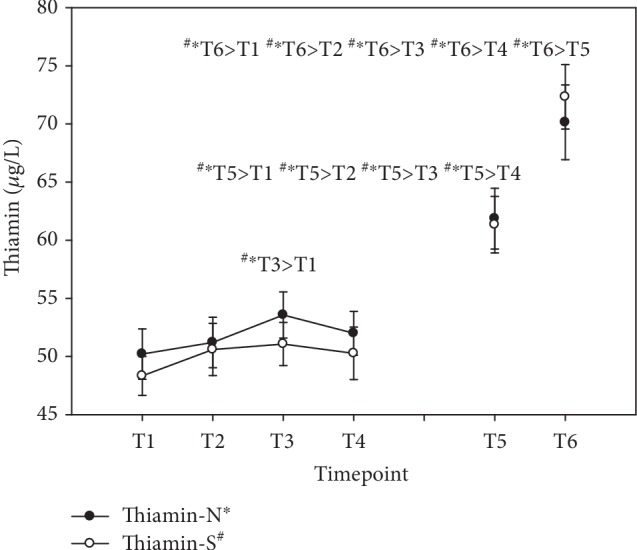
Serum thiamine levels in the follow-up for both subgroups supplemented with natural (filled circles) or synthetic (open circles) B vitamins. Blood sampling: T1 = baseline—immediately before the first supplement; T2 = 1.5 h—subsequent to the first supplementation; T3 = 4 h; T4 = 7 h; T5 = 6 weeks (end of supplementation); and T6 = 8 weeks (washout period) from baseline. Data are presented as mean values ± standard error. Significant differences (*p* < 0.05) were indicated at the respective timepoints for natural supplements (^∗^) and synthetic supplements (^#^).

In group N, supplied with the natural source of vitamin B complex, and in group S, supplied with the synthetic vitamin B complex, both bioavailability and storage capacity were very similar with respect to vitamin B_1_ serum levels. In the course of the first day, a marginal peak level (+7% N; +6% S) was achieved 4 hours after the first ingestion at T3 (see Figure 3). There was significant enrichment of serum thiamine levels at the end of vitamin B supplementation at T5 (+23% N; +27% S), which increased even further during the washout period until the final stage of the study at T6 (+40% N; +50% S), indicating an accumulation. Results were expressed as *μ*g/L (mean ± standard error).

### 3.3. Riboflavin (Vitamin B_2_)

**Figure 4:**
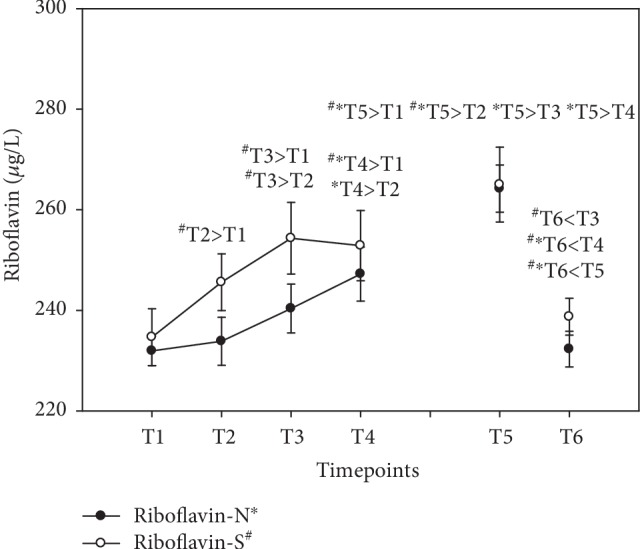
Serum riboflavin levels in the follow-up for both subgroups supplemented with natural (filled circles) or synthetic (open circles) B vitamins. Blood sampling: T1 = baseline—immediately before the first supplement; T2 = 1.5 h—subsequent to the first supplementation; T3 = 4 h; T4 = 7 h; T5 = 6 weeks (end of supplementation); and T6 = 8 weeks (washout period) from baseline. Data are presented as mean values ± standard error. Significant differences (*p* < 0.05) were indicated at the respective timepoints for natural supplements (^∗^) and synthetic supplements (^#^).

Serum levels of vitamin B_2_ increased both as a short-term effect after a single treatment (+7% N; +8% S) at T4 and as long-term effects over 6 weeks of supplementation, with a peak level at T5 in both groups (+14% N; +13% S) (see Figure 4). Unlike vitamin B_1_, we saw no storage capacity for riboflavin as vitamin B_2_ levels declined to baseline values after the washout period at T6. Results were expressed as *μ*g/L (mean ± standard error).

### 3.4. Pyridoxine (Vitamin B_6_)

**Figure 5:**
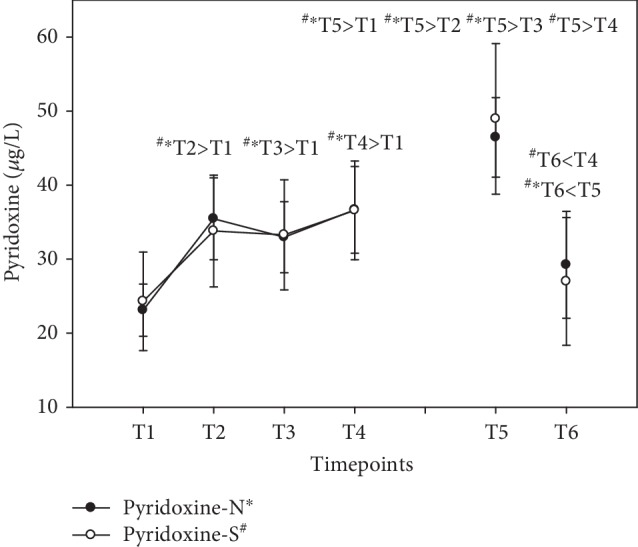
Serum pyridoxine levels in the follow-up for both subgroups supplemented with natural (filled circles) or synthetic (open circles) B vitamins. Blood sampling: T1 = baseline—immediately before the first supplement; T2 = 1.5 h—subsequent to the first supplementation; T3 = 4 h; T4 = 7 h; T5 = 6 weeks (end of supplementation); and T6 = 8 weeks (washout period) from baseline. Data are presented as mean values ± standard error. Significant differences (*p* < 0.05) were indicated at the respective timepoints for natural supplements (^∗^) and synthetic supplements (^#^), respectively.

Vitamin B_6_ serum levels increased immediately in both groups after a single dose, as early as T2, 1.5 hours after the first ingestion of the compound (+53% N; +39% S) and remained constant for the rest of the first day until T4. Vitamin B_6_ levels were enriched, with peak levels at the end of the supplementation period (T5) in both groups (+101%) and a further decline during the washout period at T6 (+26% N; +11% S—compared to baseline) (see Figure 5). Results were expressed as *μ*g/L (mean ± standard error).

### 3.5. Folic Acid (Vitamin B_9_)

**Figure 6:**
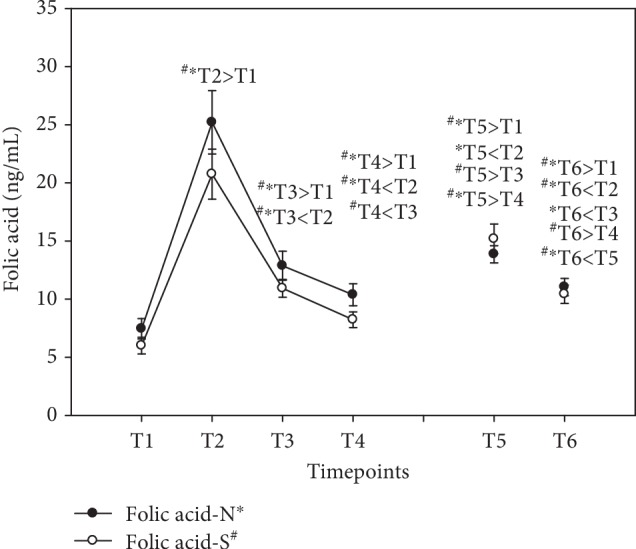
Serum folic acid levels in the follow-up for both subgroups supplemented with natural (filled circles) or synthetic (open circles) B vitamins. Blood sampling: T1 = baseline—immediately before the first supplement; T2 = 1.5 h—subsequent to the first supplementation; T3 = 4 h; T4 = 7 h; T5 = 6 weeks (end of supplementation); and T6 = 8 weeks (washout period) from baseline. Data are presented as mean values ± standard error. Significant differences (*p* < 0.05) were indicated at the respective timepoints for natural supplements (^∗^) and synthetic supplements (^#^).

These levels were lowest at baseline in both groups. Serum folic acid levels peaked as early as at T2 (+238% N; +246% S), followed by a steady decline during day 1. At the end of the supplementation period at T5, folic acid levels had significantly increased compared to baseline levels (+86% N; +153% S), though not as high as at T2, indicating the time for the best bioavailability of vitamin B_9_. Although folic acid levels were higher at T6 than at baseline, they declined significantly during the washout period between T5 and T6 (see Figure 6). Results were expressed as ng/mL (mean ± standard error).

### 3.6. Cobalamin (Vitamin B_12_)

**Figure 7:**
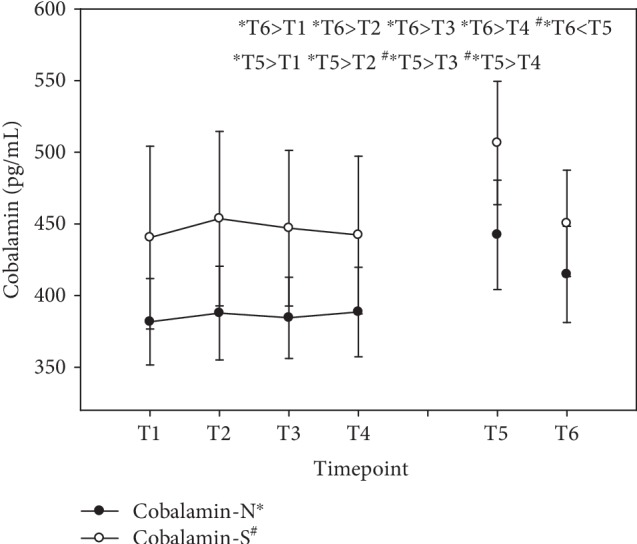
Serum cobalamin levels in the follow-up for both subgroups supplemented with natural (filled circles) or synthetic (open circles) B vitamins. Blood sampling: T1 = baseline—immediately before the first supplement; T2 = 1.5 h—subsequent to the first supplementation; T3 = 4 h; T4 = 7 h; T5 = 6 weeks (end of supplementation); and T6 = 8 weeks (washout period) from baseline. Data are presented as mean values ± standard error. Significant differences (*p* < 0.05) were indicated at the respective timepoints for natural supplements (^∗^) and synthetic supplements (^#^).

Vitamin B complex supplementation failed to show short-term effects on serum cobalamin levels with no significant changes during day 1 but with significant increase in vitamin B_12_ at T5 (+16% N; +15% S). It should be noted that group S failed to become statistically significant compared to baseline levels, although serum levels increased by 15%. The group with synthetic vitamin B only became significant compared to T3 and T4. Although cobalamin levels during the washout period were lower at T6 than at T5, serum levels of vitamin B_12_ were still higher in group N compared to baseline (+9%) and during the first day of observation, in contrast to group S, where serum levels had decreased to baseline levels at T6 (see Figure 7). Results were expressed as pg/mL (mean ± standard error).

### 3.7. Total Homocysteine (tHcy)

**Figure 8:**
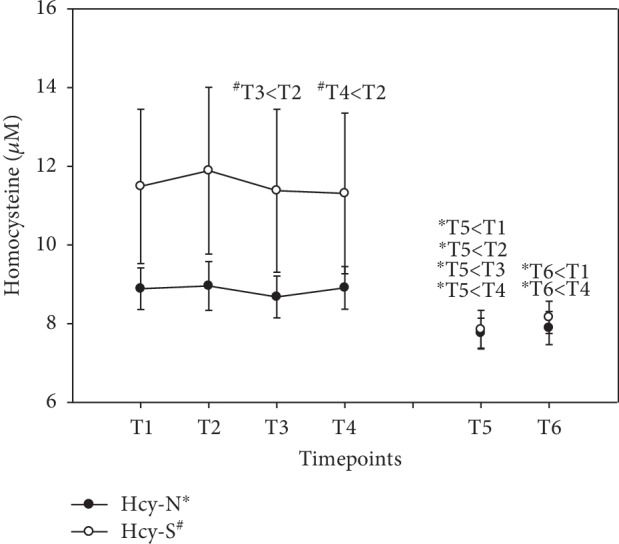
Serum homocysteine levels in the follow-up for both subgroups supplemented with natural (filled circles) or synthetic (open circles) B vitamins. Blood sampling: T1 = baseline—immediately before the first supplement; T2 = 1.5 h—subsequent to the first supplementation; T3 = 4 h; T4 = 7 h; T5 = 6 weeks (end of supplementation); and T6 = 8 weeks (washout period) from baseline. Data are presented as mean values ± standard error. Significant differences (*p* < 0.05) were indicated at the respective timepoints for natural supplements (^∗^) and synthetic supplements (^#^).

With vitamin B, tHcy had decreased significantly at the end of supplementation (T5) in group N (-13%). Even after the washout period, tHcy remained low (-11%) compared to baseline, but again, exclusively in group N (see Figure 8). Results were expressed in *μ*M (mean ± standard error).

### 3.8. Total Antioxidant Capacity (TAC)

**Figure 9:**
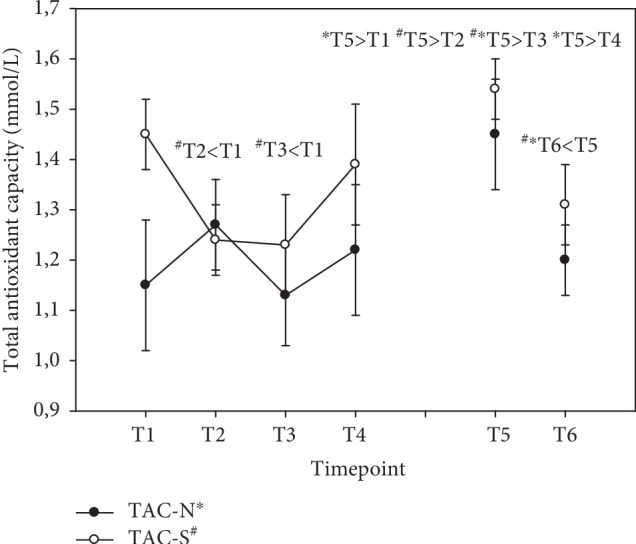
Serum total antioxidants in the follow-up for both subgroups supplemented with natural (filled circles) or synthetic (open circles) B vitamins. Blood sampling: T1 = baseline—immediately before the first supplement; T2 = 1.5 h—subsequent to the first supplementation; T3 = 4 h; T4 = 7 h; T5 = 6 weeks (end of supplementation); and T6 = 8 weeks (washout period) from baseline. Data are presented as mean values ± standard error. Significant differences (*p* < 0.05) were indicated at the respective timepoints for natural supplements (^∗^) and synthetic supplements (^#^).

During day 1 (T1-T4), the total antioxidant status in group N was unaffected after supplementation with vitamin B complex. Unexpectedly, serum TAC decreased in group S at T2 and T3, i.e., -15% and -16%, respectively. At the end of the supplementation period at T5, the antioxidant status increased significantly compared to baseline levels exclusively with peak levels in group N (+26%), in contrast to group S (+6%), where baseline levels were not significantly different. During the washout period, the antioxidant status declined to baseline levels in both groups (see Figure 9). Results were expressed as mmol/L (mean ± standard error).

### 3.9. Endogenous Peroxidase Activity (EPA)

**Figure 10:**
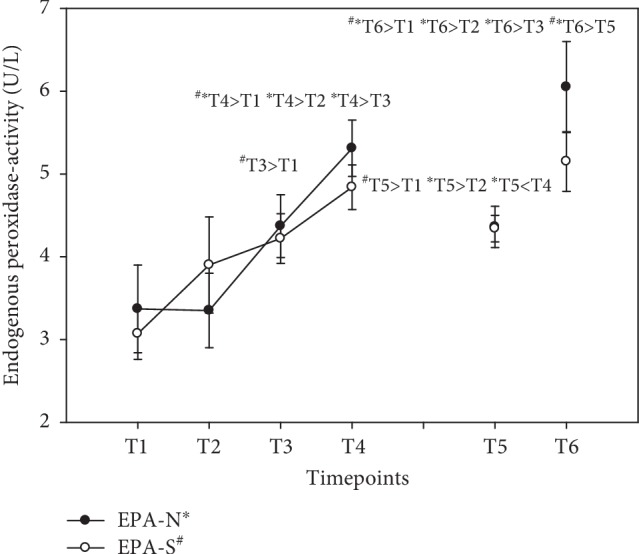
Serum endogenous peroxidase activity in the follow-up for both subgroups supplemented with natural (filled circles) or synthetic (open circles) B vitamins. Blood sampling: T1 = baseline—immediately before the first supplement; T2 = 1.5 h—subsequent to the first supplementation; T3 = 4 h; T4 = 7 h; T5 = 6 weeks (end of supplementation); and T6 = 8 weeks (washout period) from baseline. Data are presented as mean values ± standard error. Significant differences (*p* < 0.05) were indicated at the respective timepoints for natural supplements (^∗^) and synthetic supplements (^#^).

There was a linear increase in peroxidase activity in both groups during the first day, reaching peak levels at T4 (+58%) in both groups, i.e., 7 hours after the first ingestion of the vitamin B complex. At the end of the supplementation period at T5, activity was still increased in both groups (+29% N; +41% S)—reaching statistical significance solely in S—although peak levels at T4 were not attained. After the washout period at T6, there was a further increase in peroxidase activities in both groups as well (+80% N; +68% S—compared to baseline). For details, see Figure 10. Results were expressed as U/L (mean ± standard error).

### 3.10. Total Oxidant Capacity (TOC)

**Figure 11:**
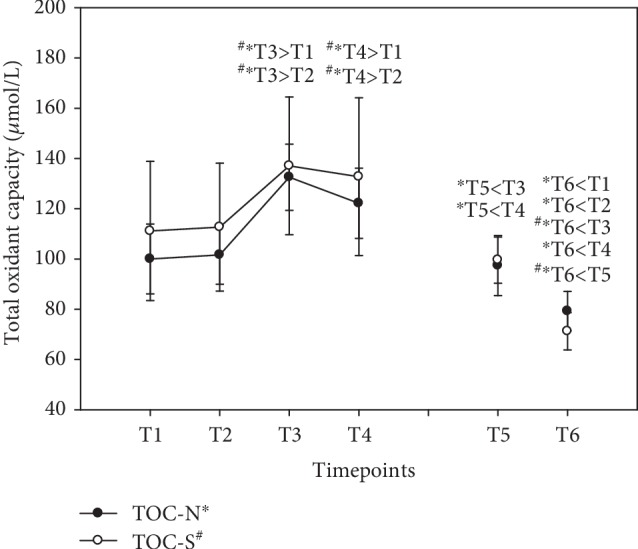
Serum total peroxides in the follow-up for both subgroups supplemented with natural (filled circles) or synthetic (open circles) B vitamins. Blood sampling: T1 = baseline—immediately before the first supplement; T2 = 1.5 h—subsequent to the first supplementation; T3 = 4 h; T4 = 7 h; T5 = 6 weeks (end of supplementation); and T6 = 8 weeks (washout period) from baseline. Data are presented as mean values ± standard error. Significant differences (*p* < 0.05) were indicated at the respective timepoints for natural supplements (^∗^) and synthetic supplements (^#^).

Total serum peroxides in both groups were hardly affected during the supplementation period, with no significant differences for this oxidative stress biomarker. The increase at T3 and T4 is attributable to fasting because the subjects had no food before blood sampling at T3. Nevertheless, there was a decrease in total peroxides in both groups after the washout period at T6, although they only fell below baseline in group N (Figure 11). Peroxide levels were specified as *μ*mol/L (mean ± standard error).

### 3.11. Polyphenols (PPm)

Serum polyphenols did not change significantly. Results were expressed as mmol/L. Mean and standard error for each B vitamin and biomarker are shown in Table 2.

## 4. Discussion

This study is aimed at comparing natural versus synthetic vitamin B in healthy subjects, with respect to bioavailability of each component as well as their effects on homocysteine and oxidative stress. A full range of B vitamins is necessary, because even in developed countries, large segments of the population, such as the elderly, have dietary vitamin B deficiencies [23].

Our main result indicated a comparable bioavailability of natural and synthetic B vitamins, and we recorded no statistically significant differences between the two groups at any timepoint. By supplementation with vitamin B (whose composition was determined by the natural occurrence in quinoa seedlings), serum levels for each B vitamin became significantly increased in both groups. For example, vitamin B_6_ and B_9_ serum levels were doubled at the end of supplementation. However, we have found some differences between the natural and synthetic sources, over time, i.e., a more sustainable effect of natural B vitamins in the case of serum cobalamin, with elevated levels not only at the end of the supplementation period but also sustained after the washout period; there was also a significant decrease of homocysteine and a significant increase of the antioxidant capacity.

Vitamin B_1_ increased significantly after a 6-week supplementation with a further increase during the washout period in both groups. A doubling of serum levels during the washout period was observed exclusively with thiamine. If this effect is already apparent in healthy people, then it is even more valuable, e.g., in older people [24] whose poor vitamin B_1_ metabolism leads to processing deficits and further to a lack of vitamin B_1_ in the brain [25]. Sufficient supply is also required among others, for children [26], celiac disease patients [27], or alcoholics [28].

Serum levels of riboflavin in our participants increased with the diurnal cycle as well as in the long run. However, constant supplementation was a prerequisite for proper serum levels as indicated by a decrease during the washout period. Hypertension is one of the major risk factors for stroke and cardiovascular disease. Riboflavin is a potent B vitamin to reduce blood pressure significantly in individuals with this genetic polymorphism [29], and in combination with folic acid, a significant decrease of homocysteine was achieved [30]. This again underlines the interaction between the individual B vitamins. In a group of 114 healthy adults, 77% had a suboptimal status for vitamin B_2_ with a harmful association in current smokers [31]. The importance of vitamin B_2_ manifests among others in the case of noninsulin-dependent diabetes mellitus, in which a significant deficiency of riboflavin has been found [32].

Oral ingestion of both natural and synthetic vitamin B complex doubled the serum level of B_6_ after a 6-week supplementation period. It should be emphasized that vitamin B_6_ can only be enriched with continuous supplementation because it declines within the 2-week washout period to nearly the baseline level. On the other hand, even a single dose was able to enrich the serum level by more than 50% within the diurnal cycle. It is noteworthy that despite increased baseline of vitamin B_6_ serum levels in both groups (N = 23.11 *μ*g/L ± 3.52 SE; S = 24.31 *μ*g/L ± 6.66 SE), which were above normal range (3.6-18 *μ*g/L), these concentrations even doubled at the end of the supplementation period. A proper supply of vitamin B_6_ was linked to better mental health because the biosynthesis of the neurotransmitters epinephrine, dopamine, and serotonin depends on vitamin B_6_-dependent enzymes [33]. This is consistent with the “Coronary Artery Risk Development in Young Adults (CARDIA) study,” which indicated that higher intake of B vitamins supported better cognitive function in midlife [34]. The importance of pyridoxine can be illustrated by the protection it affords from aortic lesions and from homocysteine-induced atherosclerosis due to its antioxidative capacity [35] based on reduction of homocysteine with folic acid and cobalamin [36, 37].

The serum level of folate increased immediately after the first ingestion with the peak after just 1.5 hours. Then, it decreased steadily with the diurnal cycle. Beside the peak at T2, serum levels of folate were higher than baseline at the end of supplementation and even after the washout period in both groups. This is consistent with the results of Verlinde et al. [38], who reported a maximum serum folate response in healthy men between 0.5 and 1.5 hours after folic acid supplementation with a significant improvement of serum folate concentrations during a cosupplementation with vitamin C. Stanger et al. [39] published a homocysteine lowering effect with 5 mg folic acid and a significant improvement of peak reactivity of resistance vessels in coronary artery disease (CAD) patients without any effect on the total antioxidant status. Interestingly, in subjects with lower homocysteine baseline levels, with a median reduction of less than 2 *μ*M, the total antioxidant status was significantly increased whereas the peak reactivity of resistance vessels was not affected. This effect indicates biphasic behaviour of folic acid: in subjects with increased homocysteine levels, folic acid is primarily responsible for a decrease in homocysteine with a simultaneous improvement in peak reactivity of resistance vessels. In contrast, in subjects with almost normal homocysteine levels, this “first aid action” was not necessary, and accordingly, the effect of folic acid was instantly directed to improve the total antioxidant capacity. Additional health-promoting effects for vitamin B_9_ were reported as preventing stroke in hypertensive adults with elevated total cholesterol levels [40], reducing the prevalence of neural tube defect at birth [41] and protecting against atherosclerosis [42, 43].

Vitamin B_12_ acts in the one-carbon transfer through methylation and is essential for humans with respect to nucleic acid, amino acid, and fatty acid metabolic pathways. Reasons for hypovitaminosis include genetic defects, malabsorption, or inadequate diet. Vegans may have an advantage with respect to BMI, glucose, and cholesterol compared to omnivores but plants do not contain cobalamin. If the supply of cobalamin is inadequate, these advantages are nullified. The daily requirement of B_12_ is 2.4 *μ*g, but higher doses can be suggested for vegans with a low B_12_ status, who had not been taking supplements over a long period of time [44, 45]. In our assay, serum cobalamin concentrations increased significantly—more sustainably in the group ingesting the natural source—with elevated levels not only at the end of the supplementation period but also with a maintenance capacity after the washout period. This finding is consistent with a report by Matte et al. [46], indicating that the natural source of vitamin B_12_ in cow's milk is superior to a synthetic supplement. Furthermore, a supplementation study with low-dose vitamin B in healthy elderly persons indicated a more pronounced effect in the reduction of cardiovascular disease risk. This vitamin B-associated effect only became apparent after 12 months of supplementation, while there was no significant difference from placebo at 6 months. This positive effect as well as an increase in HDL cholesterol disappeared within the washout period, i.e., 6 months after the end of supplementation, indicating that the associated benefits depend on a continuous supply [23].

Homocysteine levels were reported to be increased in the elderly [47]. Subjects with posttraumatic stress disorders suffer from increased homocysteine levels and are more likely to develop cardiovascular disease, whereby the severity of the illness was associated with homocysteine [48]. In our study, homocysteine was decreased in the group that took the natural vitamin B complex, possibly because of the range of dispersion in the group taking synthetic B vitamins at the beginning of the study. This is remarkable with respect to the comparatively low vitamin B concentrations as compared to other studies [13, 49]. The mere fact that the study subjects were healthy should imply an adequate antioxidant supply. We in fact observed an increased serum TAC level at the end of supplementation (T5) in the case of the natural vitamin B complex and a further decline during the washout period. It is worth mentioning that the mean value of group *N* was below the cut-off (≥1.3 mmol/L) for TAC at baseline (N = 1.15 mmol/L ± 0.13 SE; S = 1.45 mmol/L ± 0.07 SE). This is an indication that natural vitamin B complex might be superior to synthetic vitamin B complex for the antioxidant system and in the degradation of tHcy. When there is no replenishment, e.g., during the washout period, the system switches back to first aid action to decompose homocysteine. Endogenous antioxidants increased both in the diurnal cycle and in the long-term observation period, especially during the washout phase. This effect was associated with less oxidative stress as reflected in a significant decrease in total peroxides. Total peroxides were previously shown to decrease in patients undergoing coronary artery bypass graft surgery who were given a vitamin C and vitamin E cocktail [50]. Consequently, vitamin B complex seems to be a key player in the redox cycle, thus preventing oxidative stress.

The main weakness of this pilot study was the exclusion of the crossover part due to carryover effects in the majority of biomarkers. This effect was underestimated since B vitamins are water-soluble and rapidly metabolized, so that any excess should be excreted with the urine. Another weakness was the exclusion of niacin and pantothenic acid serum levels due to limited funding. Since by definition a pilot study has a limited number of cases, this might account for the failure of some trends between the groups to achieve significance. These trends would nonetheless warrant a suitably powered clinical trial.

## 5. Conclusions

This clinical double-blind pilot study focused on systemic alterations of serum vitamin B levels in healthy subjects in response to a vitamin B supplementation (natural vitamins versus synthetic forms), in the range of about 2.5 times above the recommended daily allowance for six weeks, and a washout period for another two weeks. With regard to bioavailability, both groups showed remarkable increases in serum levels of each B vitamin at the end of supplementation. The increase in serum vitamin B levels was associated with improved antioxidant status, with both nonenzymatic and enzymatic antioxidants inversely related to a decrease in oxidative stress (including homocysteine). It should be emphasized that these effects were attainable through a composition of B vitamins based on a pattern found in quinoa germlings. Although there were a few favourable trends in the group supplemented with the natural source, e.g., a decrease of homocysteine and a long-lasting effect for cobalamin and peroxidase activity even after the washout period, the differences between the groups did not achieve significance.

## Figures and Tables

**Table 1:** Basic characteristics.

	Group A	Group B	*p* values
Females/males (*n*, %)	11 (78.6)/3 (21.4)	10 (66.7)/5 (33.3)	0.682
Age (years)	37.71 ± 3.72*a*	37.87 ± 3.64	0.977
Body mass index (kg/m^2^)	22.49 ± 0.57	22.35 ± 0.75	0.890
Thiamin (*μ*g/L)	50.21 ± 2.16	48.33 ± 1.68	0.494
Riboflavin (*μ*g/L)	231.93 ± 2.93	234.67 ± 5.65	0.677
Pyridoxine (*μ*g/L)	23.11 ± 3.52	24.31 ± 6.66	0.238
Folic acid (ng/mL)	7.45 ± 0.88	6.00 ± 0.72	0.211
Cobalamin (pg/mL)	381.71 ± 30.13	440.47 ± 63.73	0.914
TAC (mmol/L)	1.15 ± 0.13	1.45 ± 0.07	0.056
PPm (mmol/L)	9.88 ± 0.09	9.57 ± 0.09	0.024^∗^
EPA (U/L)	3.37 ± 0.53	3.07 ± 0.31	0.615
TOC (*μ*mol/L)	100.00 ± 13.92	111.17 ± 27.72	0.868
Homocysteine (*μ*M)	8.89 ± 0.53	11.49 ± 1.96	0.407

^a^Data are expressed as mean ± SE. ^∗^Significantly different *p* < 0.05.

**Table 2:** Serum concentrations for B vitamins, homocysteine, antioxidants, peroxidase activity, peroxides, and polyphenols.

	T1 (mean ± SE)	T2 (mean ± SE)	T3 (mean ± SE)	T4 (mean ± SE)	T5 (mean ± SE)	T6 (mean ± SE)
Thiamine (*μ*g/L)						
N	50.21 ± 2.16	51.21 ± 2.17	53.57 ± 1.99	52.00 ± 1.89	61.86 ± 2.62	70.14 ± 3.22
S	48.33 ± 1.68	50.60 ± 2.25	51.07 ± 1.86	50.27 ± 2.26	61.33 ± 2.43	72.33 ± 2.78
Riboflavin (*μ*g/L)						
N	231.93 ± 2.93	233.86 ± 4.79	240.36 ± 4.85	247.21 ± 5.37	264.21 ± 4.71	232.29 ± 3.55
S	234.67 ± 5.65	245.60 ± 5.65	254.33 ± 7.13	252.87 ± 6.98	265.00 ± 7.44	238.73 ± 3.66
Pyridoxine (*μ*g/L)						
N	23.11 ± 3.52	35.45 ± 5.52	32.96 ± 4.80	36.66 ± 5.85	46.44 ± 5.37	29.23 ± 7.21
S	24.31 ± 6.66	33.80 ± 7.55	33.28 ± 7.44	36.59 ± 6.67	48.94 ± 10.17	26.99 ± 8.62
Folic acid (ng/mL)						
N	7.45 ± 0.88	25.21 ± 2.72	12.87 ± 1.25	10.38 ± 0.95	13.86 ± 0.74	11.04 ± 0.75
S	6.00 ± 0.72	20.75 ± 2.15	10.93 ± 0.77	8.23 ± 0.68	15.18 ± 1.28	10.43 ± 0.79
Cobalamin (pg/mL)						
N	381.71 ± 30.13	387.79 ± 32.73	384.50 ± 28.31	388.57 ± 31.20	442.36 ± 38.15	414.79 ± 33.59
S	440.47 ± 63.73	453.67 ± 60.86	447.00 ± 54.23	442.27 ± 55.04	506.47 ± 42.98	450.33 ± 37.08
Homocysteine (*μ*mol/L)						
N	8.89 ± 0.53	8.96 ± 0.62	8.68 ± 0.53	8.91 ± 0.54	7.76 ± 0.38	7.89 ± 0.42
S	11.49 ± 1.96	11.89 ± 2.12	11.38 ± 2.07	11.31 ± 2.04	7.85 ± 0.49	8.16 ± 0.41
Total antioxidants (mmol/L)						
N	1.15 ± 0.13	1.27 ± 0.09	1.13 ± 0.10	1.22 ± 0.13	1.45 ± 0.11	1.20 ± 0.07
S	1.45 ± 0.07	1.24 ± 0.07	1.23 ± 0.10	1.39 ± 0.12	1.54 ± 0.06	1.31 ± 0.08
Endogenous peroxidase activity (U/L)						
N	3.37 ± 0.53	3.35 ± 0.45	4.37 ± 0.38	5.31 ± 0.34	4.36 ± 0.25	6.05 ± 0.55
S	3.07 ± 0.31	3.90 ± 0.58	4.22 ± 0.30	4.84 ± 0.27	4.34 ± 0.16	5.15 ± 0.36
Total peroxides (*μ*mol/L)						
N	100.00 ± 13.92	101.64 ± 11.65	132.55 ± 13.18	122.18 ± 13.99	97.36 ± 11.97	79.27 ± 7.80
S	111.17 ± 27.72	112.67 ± 25.47	137.08 ± 27.44	132.75 ± 31.41	99.58 ± 9.19	71.25 ± 7.42
Polyphenols (mmol/L)						
N	9.88 ± 0.09	9.73 ± 0.12	9.97 ± 0.10	9.72 ± 0.08	9.49 ± 0.09	9.73 ± 0.08
S	9.57 ± 0.09	9.77 ± 0.17	9.81 ± 0.12	9.54 ± 0.11	9.46 ± 0.11	9.54 ± 0.14

## Data Availability

The data used to support the findings of this study are available from the corresponding author upon request.

## Supplementary Materials

Supplementary Materials CONSORT 2010 checklist of information to include when reporting a randomized trial^∗^.
